# COMMUNITY-BASED DEMENTIA SCREENING AND SUPPORTIVE SERVICES

**DOI:** 10.1093/geroni/igae098.3071

**Published:** 2024-12-31

**Authors:** Traci Wilson, Mary Ek

**Affiliations:** USAging, Washington, District of Columbia, United States; USAging, Washington, District of Columbia, United States

## Abstract

Early detection of dementia is important to help consumers and caregivers access information, treatment and supportive services. The majority of people living with dementia (PLWD) live at home and in the community. Community-based aging services providers, such as Area Agencies on Aging (AAAs), are positioned to play a critical role in screening for cognitive impairment or dementia and referring individuals for medical cognitive assessments. Past surveys have shown that AAAs provide specialized services for PLWD and caregivers, but little is known about the prevalence of screenings and the process for referrals. This poster shares findings from a 2024 survey of AAAs about their dementia screening and supports (n=180, response rate 28%) describing the formal and informal cognitive screening, assessment, and referral services that AAAs provide, the feedback available to consumers and the agency after referrals are made, and the types of services and supports available that are tailored specifically for PLWD. The most common screening or assessment service is referrals to external sources of information (84%), followed by agency and provider staff observing for signs and symptoms of dementia (55%), referring individuals to medical cognitive assessments (43%), and conducting screenings (31%). The results demonstrate that AAAs are involved in a variety of dementia-related screenings, services, and interventions.

